# Management of Petrous and Tentorial Dural Arteriovenous Fistulas: A Systematic Review

**DOI:** 10.7759/cureus.73701

**Published:** 2024-11-14

**Authors:** Injam Ibrahim Sulaiman, Ali Hassan Baker, Azhin Shafeeq, Mohammed Bani Saad, Mustafa Ismail

**Affiliations:** 1 Department of Surgery, Hawler Medical University, College of Medicine, Erbil, IRQ; 2 Department of Neurosurgery, Hawler Teaching Hospital, Erbil, IRQ; 3 Department of Surgery, Al-Kindy Teaching Hospital, Baghdad, IRQ; 4 Department of Surgery, College of Medicine, University of Baghdad, Baghdad, IRQ; 5 Department of Surgery, Baghdad Teaching Hospital, Medical City Complex, Baghdad, IRQ

**Keywords:** digital subtraction angiography, endovascular embolization, intracranial hemorrhage, microsurgical treatment, petrous davfs, tentorial davfs

## Abstract

The petrous and tentorial dural arteriovenous fistulas are vascular malformations that are very infrequent but highly aggressive, with a significant risk of intracranial hemorrhage and neurological deficits. Optimal management remains one of the most debated subjects, with various series reporting endovascular and microsurgical approaches. Therefore, this systematic review aims to assess the efficacy, safety, and outcomes of different treatment modalities of petrous and tentorial dural arteriovenous fistulas (DAVFs) based on clinical presentation, imaging techniques, treatment outcome, and complications arising in the course of their treatment. A systematic review based on Preferred Reporting Items for Systematic Reviews and Meta-Analyses (PRISMA) was carried out, which aimed to identify literature regarding both petrous and tentorial DAVFs. Major databases, including PubMed and Scopus, are searched using various related terms. Patient demographics as well as clinical presentations of patients with petrous and tentorial DAVFs were abstracted concerning imaging modalities, approaches adopted for their treatment, and the eventual outcome. The quality of studies was assessed using the Newcastle-Ottawa Scale, and data were synthesized through descriptive analysis.

A total of 14 studies involving 198 patients were included. The mean patient age ranged from 38 to 59.8 years, with a male predominance (78%). Clinical presentations varied from headaches and tinnitus to life-threatening intracranial hemorrhage. Digital subtraction angiography (DSA) was the gold standard for diagnosis, while MRI and CT were useful adjuncts in assessing hemorrhage and venous drainage. Endovascular embolization using Onyx achieved complete obliteration in the majority of cases, though recurrence was noted in fistulas with complex arterial supply. Microsurgical approaches, particularly in cases where endovascular treatment was insufficient, demonstrated high cure rates with low recurrence. Complications included cranial nerve palsies and, in rare cases, arterial or venous rupture. Mortality was low, with a case fatality rate of 0%-15.4% across the studies. Both endovascular and microsurgical treatments are effective for managing petrous and tentorial DAVFs, though microsurgery provides superior results in complex or recurrent cases. A combination of embolization and surgery offers the best chance for durable fistula obliteration. Early diagnosis and individualized treatment plans guided by advanced imaging are critical for optimizing patient outcomes.

## Introduction and background

Dural arteriovenous fistulas (DAVFs) represent a rare but clinically important subgroup of intracranial vascular malformations, with an approximate percentage of intracranial arteriovenous malformations comprising 10%-15% [1]. Of the fistulas at the petrous apex and tentorial regions, the anatomy is complex and has essential neurovascular structures in proximity. These fistulas are characterized by typical anomalous arteriovenous shunts within the dura mater and directly drain arterial blood into the venous system, bypassing an intervening capillary network. This pattern of anomalous blood flow is fraught with extremely significant risks, including venous hypertension, intracranial hemorrhage, and progressive neurological deficits [2].

Although venous thrombosis, trauma, and congenital vascular anomalies are some of the factors that have been implicated in the pathogenesis of DAVFs, they remain incompletely understood [3]. Many DAVFs are asymptomatic, while others present aggressively, with symptoms that range from pulsatile tinnitus and cranial nerve palsies to life-threatening hemorrhage. Of the DAVFs, tentorial lesions have a particular tendency toward an aggressive clinical presentation, usually presenting with cortical venous drainage that has a high propensity for hemorrhage [4].

Indeed, during the last few decades, advances in diagnostic imaging and therapy have significantly improved the diagnosis and management of such complex lesions. Digital subtraction angiography (DSA), MRI, and CT are important in establishing the correct anatomical location and the hemodynamic characteristics of the DAVFs [5]. The therapeutic options have evolved and now range from a combination of microsurgical approaches to endovascular techniques, including transarterial and transvenous embolization methods, variably demonstrating total obliteration of the fistula [6].

For that reason, a comprehensive and systematic review of the literature is required with respect to the variability in appearances presented by petrous and tentorial DAVFs in relation to symptomatology, anatomical construct, and results of treatments employed. It is intended to critically evaluate the efficacy and safety of current surgical and endovascular treatment designs underlying patient outcomes, and summarize factors associated with recurrence, and case fatality. This review synthesizes findings from recent studies in an attempt to provide clinicians with evidence-based insights into optimizing the management of these rare yet challenging vascular lesions.

## Review

Methods

Study Design

**Figure 1:**
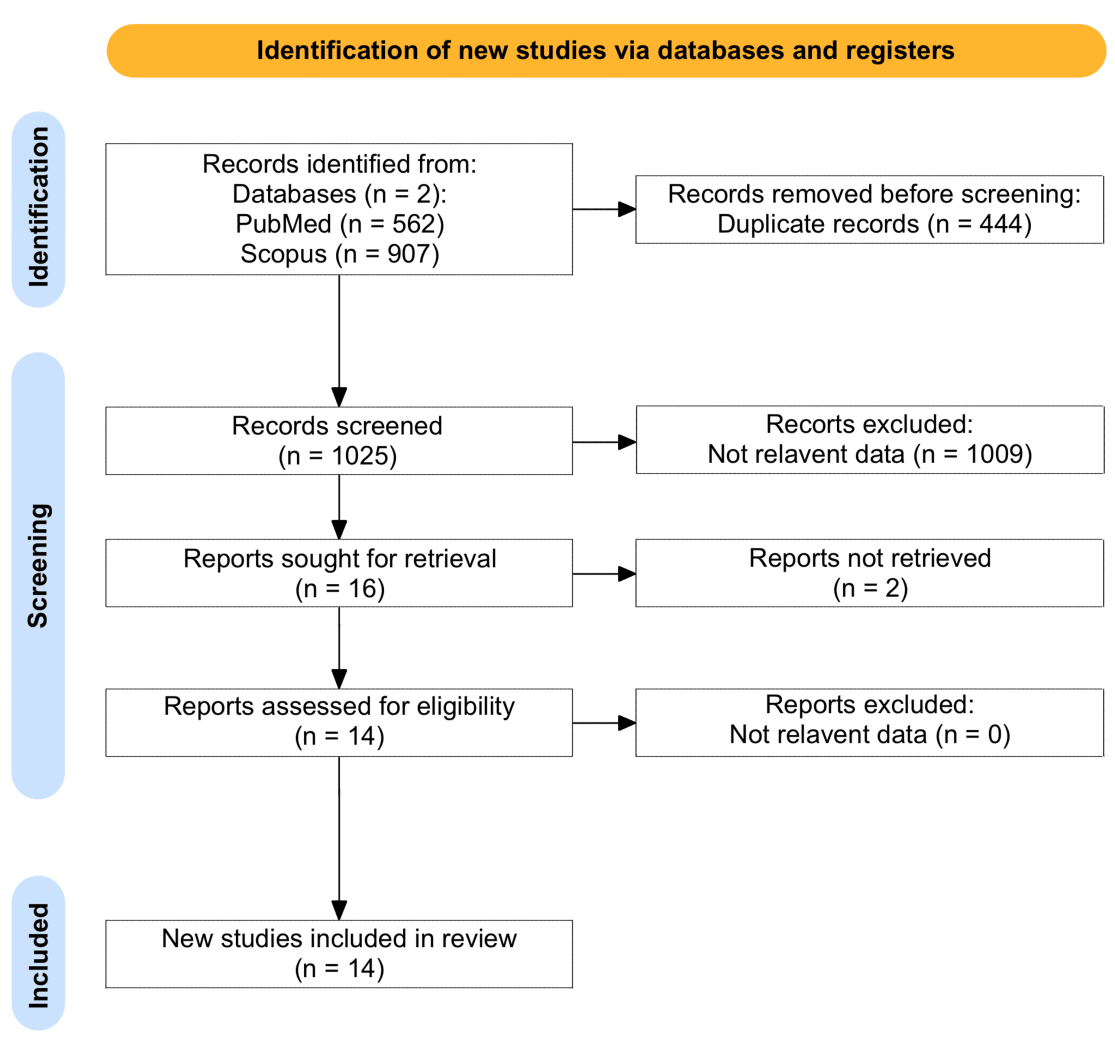
A PRISMA flowchart of the included studies PRISMA: Preferred Reporting Items for Systematic Reviews and Meta-Analyses

This systematic review has been performed according to the Preferred Reporting Items for Systematic Reviews and Meta-Analyses (PRISMA) guidelines [7] (Figure 1). The current study therefore aims to systematically analyze reported data from previous studies on DAVFs of the petrous and tentorium regarding treatment outcomes, complication rates, and recurrences. A protocol was set before the review commenced, which emphasized the study design, inclusion and exclusion criteria, methods of data extraction, data synthesis, and quality assessment methods.

Literature Search and Study Selection

An extensive literature search was performed through two major electronic databases: PubMed, and Scopus. Search terms used included "dural arteriovenous fistula," "DAVF," "petrous fistula," "tentorial fistula," "endovascular treatment," "microsurgical treatment," and "complications." Boolean operators were applied to achieve specificity regarding the results. The literature search date was conducted to include all the possible records from inception until September 2024. Additionally, reference lists of selected articles were manually examined to identify relevant studies that may have been missed. The total number of the included articles after duplicate removal was 1,025. The initial screening was based on the title and abstract, followed by a full-text review of articles that appeared to meet the inclusion criteria, which yielded 14 articles. Two independent reviewers performed the screening, and any disagreements were resolved through discussion or by involving a third reviewer. The study selection process is summarized in Figure 1. The online tool Rayyan (Rayyan Systems Inc., Cambridge, MA) was used to conduct the screening process.

Inclusion and Exclusion Criteria

Studies eligible for inclusion were those published in peer-reviewed journals, reporting on patients with petrous or tentorial DAVFs, and providing clinical outcome data related to surgical or endovascular treatments. Only studies with a minimum of five patients and follow-up data on efficacy, recurrence, and complications were considered. Exclusion criteria included case reports, editorials, review articles, studies lacking detailed treatment outcomes, or those published in languages other than English or without full-text availability. Animal- or cadaver-related articles were also excluded.

Data Extraction

Two independent reviewers extracted data from the included studies using a standardized data collection form. The variables extracted included study identifier (authors and publication year), study design, sample size, patient demographics (age and gender distribution), symptoms, imaging techniques used for diagnosis, treatment modalities (surgical or endovascular), clinical outcomes (treatment efficacy, symptom resolution, recurrence), complications, and case fatality rates. All extracted data were cross-verified for accuracy, and any discrepancies were resolved through discussion.

Data Synthesis and Quality Assessment

The extracted data were synthesized qualitatively, given the heterogeneous nature of the included studies. Descriptive statistics were used to summarize the patient demographics, clinical features, diagnostic methods, and treatment outcomes. Each selected article underwent a qualitative assessment to evaluate its contribution to the field, focusing on methodology, results, and conclusions. Cross-referencing citations within these articles helped identify additional relevant studies, ensuring a comprehensive review of the subject matter.

Results

Study Characteristics

**Figure 2:**
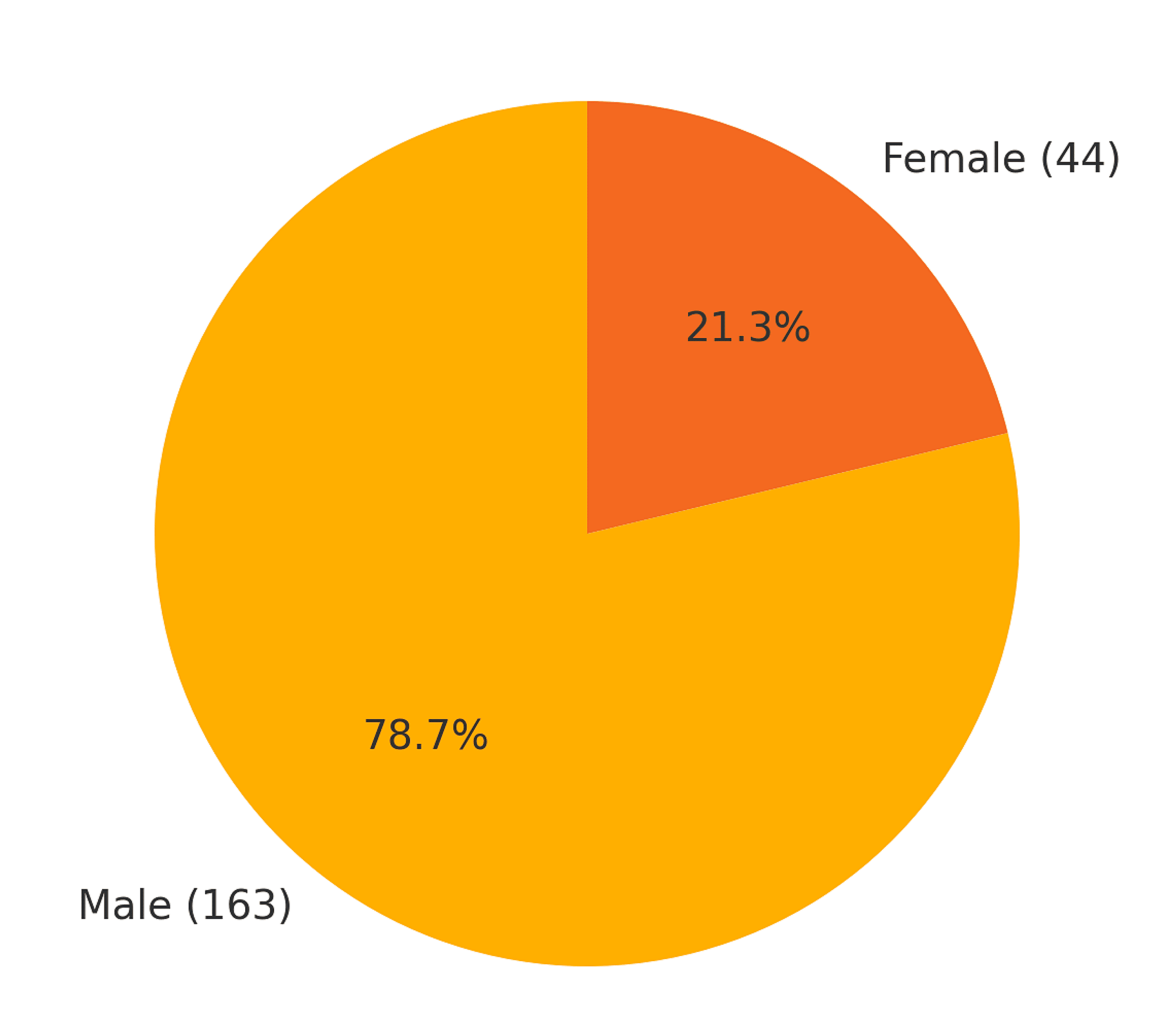
Gender distribution

**Figure 3:**
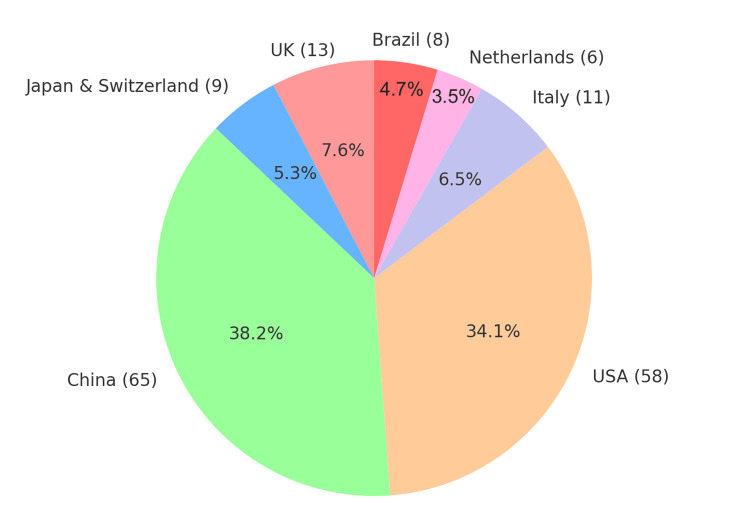
Geographical presentation of the included cases

This systematic review included 14 studies, all of which were retrospective in nature, published between 2003 and 2020. The studies were conducted in a variety of geographical locations, including China, the United States, Japan, the United Kingdom, Brazil, and the Netherlands, reflecting the global interest in the management of petrous and tentorial DAVFs. Sample sizes of the studies varied from five to 31 patients, totaling 198 patients in different studies. The details of each study are mentioned in Table 1 [1-6, 8-15]. Demographic data are represented in Table 2 and Figures 2-3.

**Table 1:** Summary of clinical characteristics, treatment approaches, and outcomes in studies on petrous and tentorial dural arteriovenous fistulas (DAVFs)

ID	Study name and publication year	Study type	Timeframe of the study	Sample size	Country	Patient age (Mean ± SD)	Gender distribution (number and percentage)	Symptoms	Imaging techniques	Surgical approach/intervention	Efficacy and outcomes of surgery	Complications	Response to treatment	Recurrence	Case fatality rate
1	Ng et al. (2003) [6]	Retrospective review	April 1986 - March 2002	18	USA	54 years (39-77)	15 males (83.3%), 3 females (16.7%)	Intracranial hemorrhage (50%), trigeminal neuralgia (11%), pulsatile tinnitus (11%), ocular symptoms (11%)	Angiography, MRI, CT	Transarterial and transvenous embolization, combined with surgery	Angiographic cure in all patients, 30-day morbidity 11%, no mortality	Trigeminal nerve palsy (21%), trochlear nerve palsy, dysphasia, hemiparesis, subarachnoid hemorrhage (1 patient)	Most patients returned to independent clinical status (Glasgow Outcome Scale 1 or 2)	None reported	0% (No deaths)
2	van Rooij et al. (2006) [14]	Retrospective series	1995 - 2005	6	Netherlands	57.3 years (37-78)	4 males (66.7%), 2 females (33.3%)	Intracerebral hemorrhage (2), pulsatile tinnitus (2), progressive tetraparesis (1), incidental (1)	Angiography, MRI, CT	Tentorial artery embolization, with techniques including glue and coil embolization	Complete occlusion in all cases, follow-up confirmed no recurrence	No complications reported	Improvement in clinical outcomes, full resolution of symptoms in most cases	None reported	0% (No deaths)
3	Zhou et al. (2007) [1]	Retrospective review	June 2002 - May 2003	5	China	38 years (25-52)	2 males (40%), 3 females (60%)	Subarachnoid hemorrhage (SAH) (40%), progressive neurological deficits (60%)	MRI, digital subtraction angiography (DSA)	Transarterial embolization and craniotomy with coagulation	Complete obliteration in all patients, no recurrence	1 case of cerebellar infarction due to embolization	Improvement in all patients	2-3 years follow-up, no recurrence	0% (No deaths)
4	Jiang et al. (2008) [9]	Retrospective series	2005 - 2008	19	China	47.5 years (23-70)	16 males (84.2%), 3 females (15.8%)	Intracranial hemorrhage, neurological deficits, headache, tinnitus	Angiography, CT	Transarterial and transvenous embolization	Complete obliteration in 13 patients, residual fistula in 6	1 case of venous rupture, 2 cases of cranial neuropathy, 1 death	Most patients had good or excellent outcomes (mRS 0-1)	None reported	5.3% (1 death due to venous rupture)
5	Lawton et al. (2008) [10]	Retrospective series	1997 - 2006	31	USA	53 years (25-87)	21 males (67.7%), 10 females (32.3%)	Intracranial hemorrhage, venous hypertension, neurological deficits	Angiography, CT, MRI	Microsurgical interruption of venous drainage, various approaches depending on fistula type	Complete obliteration in 94%, residual in 6%	Transient neurological morbidity in 4 patients, no permanent neurological morbidity, no mortality	Good outcomes (mRS 0-2) in most patients	None reported	0%
6	Huang et al. (2009) [8]	Retrospective series	2005 - 2008	14	China	50 years (37-65)	13 males (92.9%), 1 female (7.1%)	Subarachnoid hemorrhage, intraventricular hemorrhage, progressive headaches, dizziness	CT, MRI, angiography	Transarterial embolization using Onyx	Complete obliteration in 11 patients, partial in 2	1 case of vessel perforation, 1 case of microcatheter retention, 1 death due to severe infection	Symptoms resolved in most patients, follow-up showed no rebleeding or worsening	None reported	7.1% (1 death due to severe infection)
7	Pichierri et al. (2012) [2]	Retrospective review	Unknown	11	Italy	55.3 years (45-68)	9 males (81.8%), 2 females (18.2%)	Cranial nerve palsy, headache, pulsatile tinnitus, ocular symptoms	MRI, angiography	Transarterial and transvenous embolization, retrosigmoid approach	Complete cure in 9 patients, 2 required additional treatments	Cranial nerve palsy in 2 patients, no deaths	Most patients experienced symptom improvement or resolution	None reported	0% (No deaths)
8	Wajnberg et al. (2012) [15]	Retrospective series	January 2005 - July 2009	8	Brazil	53.5 years (42-70)	5 males (62.5%), 3 females (37.5%)	Subarachnoid hemorrhage (50%), intraparenchymal hemorrhage (25%), tinnitus (12.5%)	MRI, CT, angiography	Endovascular treatment (transarterial in 62.5%, transvenous in 25%, combined in 12.5%)	Complete obliteration in all patients	1 death due to pulmonary fibrosis (unrelated to the procedure)	Improvement in clinical outcomes in all surviving patients	None reported	12.5% (1 death due to pulmonary fibrosis)
9	Byrne and Garcia (2013) [3]	Retrospective review	January 2007 - September 2012	13	UK	59.8 years (45-66)	8 males (61.5%) and 5 females (38.5%)	Headache, tinnitus, visual disturbances, transient weakness, progressive confusion, intracranial hemorrhage	CT, MRI, DSA	Endovascular, surgical	5 cured, 6 subtotal occlusion, staged treatment ongoing for 1 patient, 1 cured after surgery	Procedural complications in 4 cases (arterial dissection, perforation, microcatheter rupture)	Complete symptom resolution in 3, improvement in 6, unchanged in 1	Not mentioned	15.4% (2 deaths: 1 due to arterial dissection, 1 due to infection)
10	Hatano et al. (2013) [5]	Retrospective series	1998 - 2006	9	Japan and Switzerland	52.7 years (33-70)	8 males (88.9%), 1 female (11.1%)	Headache, vertigo, facial sensory disturbances, hearing disturbances, diplopia, hemiparesis, gait disturbances, intracranial hemorrhage	CT, MRI, angiography	Subtemporal approach (8), supracerebellar approach (1)	Complete obliteration of fistulas achieved in all patients	1 case of asymptomatic temporal lobe hemorrhage	Symptoms resolved completely in most patients, with no permanent neurological deficits	Not mentioned	0%
11	Liu et al. (2014) [12]	Retrospective series	2005 - 2010	26	China	51 years (31-70)	21 males (80.8%), 5 females (19.2%)	Headache, dizziness, ataxia, pulsatile tinnitus, paralysis of cranial nerves, hemiplegia	CT, MRI, angiography	Transarterial embolization using Onyx	Complete obliteration in 17 patients, subtotal in 9	Transient paralysis of CN III in 1 patient	Significant improvement in symptoms, no rebleeding	None reported	0% (No deaths)
12	Stapleton et al. (2018) [4]	Retrospective series	August 2010 - November 2015	9	USA	58.2 years (51-67)	7 males (77.8%), 2 females (22.2%)	Venous hypertension, tinnitus, subarachnoid hemorrhage	MRI, angiography	Endovascular embolization, retrosigmoid approach	Complete occlusion in 4 patients with primary surgery, 3 recurred after embolization and required surgery	1 case of cranial nerve IV palsy, no deaths	Improved outcomes post-surgery, follow-up confirmed complete occlusion	None reported	0% (No deaths)
13	Sun et al. (2020) [13]	Retrospective series	February 2014 - June 2019	12	China	51.7 years (28-68)	10 males (83.3%), 2 females (16.7%)	Venous hypertensive myelopathy (83.3%), subarachnoid hemorrhage (16.7%)	MRI, angiography	Microsurgical treatment using indocyanine green video angiography (ICG-VA)	Complete obliteration in all cases, no recurrence reported	1 case of rebleeding post partial embolization	Most patients experienced improvement in symptoms, good to excellent outcomes	None reported	0% (No deaths)
14	Li et al. (2018) [11]	Retrospective series	2002 - 2017	26	China	46.54 years (28-73)	24 males (92.3%), 2 females (7.7%)	Nonhemorrhagic neurological defects (73.1%), subarachnoid hemorrhage (19.2%), asymptomatic (7.7%)	CT, MRI, angiography	Transarterial embolization, embolization combined with microsurgery	Complete occlusion in 21 patients, 2 patients with rebleeding post embolization	Rebleeding in 2 patients (7.7%), transient cranial nerve palsy in 1 patient	Barthel Index improved significantly in most patients	None reported	7.7% (2 deaths due to cardiopulmonary arrest post-rebleeding)

**Table 2:** Demographic overview of studies on petrous and tentorial dural arteriovenous fistulas

Metric		Value
Total sample size		207
Average age (Mean+SD)		51.79 ± 5.75 years
Gender	Male	163 individuals (78.74%)
	Female	44 individuals (21.26%)
Countries represented		UK, Japan, Switzerland, China, USA, Italy, Netherlands, Brazil

Follow-up duration for the patients varied, but most fell between six months and three years. A preponderance of the studies focused either on endovascular or microsurgery interventions, though several studies used a combination of both. The diagnostic imaging techniques included DSA, CT, and MRI, which were applied universally in the studies included for diagnostic and follow-up purposes [9,10,11]. Clinical outcomes reported in most of the included studies were treatment efficacy, resolution of symptoms, recurrence rates, complications, and mortality.

Demographic Characteristics

A total of 14 studies on the petrous and tentorial DAVFs were considered for this systematic review. Patients ranged from five in some studies to 31 in others, totaling 198 patients across the studies (Table 2, Figure 2).

Mean patient age accounted for a range from 38 years to 59.8 years; this reflects the typical middle-aged to older adult population category within the affected lesion category [3,10]. Most were males, with male representation ranging from 61.5% to 92.9% in the series. Men constituted approximately 78% of the patients in the aggregate series, while females accounted for approximately 22%. Collectively, the series also encompassed most geographic regions of the world, hence having a sizeable number of cases contributed by China, the USA, the UK, and Japan established DAVFs as a global clinical problem (Figure 3) [6, 15].

Clinical Presentation

The presentation of the petrous and tentorial DAVFs was quite variable, with symptoms ranging from nonspecific headaches to life-threatening hemorrhages. Indeed, among the series, intracranial hemorrhage was repeatedly cited as the most common presenting symptom, with an incidence as high as 50% in several cohorts, as reported by Ng et al. in 2003 [6]. Other symptoms commonly encountered included pulsatile tinnitus, progressive neurological deficits such as hemiparesis and cranial nerve palsies, vertigo, and ataxia. The above-mentioned symptoms were sometimes joined with several involved neurological signs, such as facial sensory disturbances, diplopia, and trigeminal neuralgia, which have been reported in many studies because of the contiguous location of DAVFs to critical cranial nerves. Do not forget headaches and dizziness that were common accompanying symptoms in patients without hemorrhage. In this context, Zhou et al. (2007) and Lawton et al. (2008) described the development of non-hemorrhagic neurological deficits in as many as 73% of their patients and emphasized the potential for DAVFs to be disabling even without overt hemorrhage [1,10].

Imaging Techniques

Imaging played a central role in the diagnosis and management of DAVFs. Indeed, all studies used DSA as the universal gold standard for the characteristic visualization of arteriovenous shunting. MRI and CT were also universally used for the evaluation of the extent of the lesion, associated hemorrhages, and venous drainage patterns. In fact, in studies such as Stapleton et al. (2018) [4] and Huang et al. (2009) [8], MRI was especially useful for the delineation of associated venous anomalies, while CT provided important images during the visualization of subarachnoid and intraventricular hemorrhages. In selected cases, intraoperative indocyanine green video angiography (ICG-VA) was employed in techniques that may assist in making treatment decisions and confirming obliteration of fistulas during surgeries.

Treatment Modalities

A range of different surgical and endovascular treatments were used in the studies included. The type of intervention was chosen according to the anatomical location of the fistula, its arterial feeding, and the distal extent of venous drainage involved. The most frequently performed endovascular approach was transarterial embolization, mainly with the liquid embolic agent Onyx, in eight of the included studies. Complete obliteration rates post-transarterial embolization varied; for instance, Liu et al. (2014) [12] documented successful treatment in 17 of their 26 patients, while Huang et al. (2009) [8] recorded complete obliteration in 11 out of 14 patients. This modality of treatment, despite its effectiveness, resulted in vessel perforation and/or microcatheter retention in 7.1% to 10% of the patients due to associated complications. Some cases further showed rebleeding following embolization; two such patients were recorded in the study by Li et al. [11]. This factor points out that incomplete occlusion of the fistula is still associated with risks.

Several of them employed the technique of transvenous embolization, sometimes combined with transarterial approaches. This technique showed good results, especially in cases where direct access to the venous side of the fistula was possible. Some studies, like those by Jiang et al. (2008) [9] and Ng et al. (2003) [6], reported high obliteration of the fistula with combined approaches, although complications such as rupture of the vein and cranial nerve palsies occurred in a minority of patients. Microsurgical techniques are often utilized where endovascular approaches are inadequate or anatomically not feasible. Surgical approaches often included direct disruption of the venous outflow or coagulation of the fistula itself; series such as those of Lawton et al. (2008) [10] and Stapleton et al. (2018) [4] reported near-complete obliteration rates of 94% and 100%, respectively.

Some cohort studies preferred a surgical approach for DAVFs with complex arterial supply, like those that were predominantly supplied by the meningohypophyseal trunk, which is generally regarded as notoriously difficult to reach endovascularly. Three patients from Stapleton et al.'s 2018 series who received embolization for superior petrosal sinus DAVFs went on to require surgery because of recurrence. The surgical cure rate after direct surgery was 100%, and none of the patients with surgery demonstrated permanent neurological deficits [4].

Efficacy and Outcomes of Treatment

The overall efficacy was different for each treatment modality in the various studies. Most series reported complete occlusion of a majority of their fistulas, ranging from 78% to 100%, depending on the series and modality of treatment. There were studies, such as those by Wajnberg et al. (2012) [15] and van Rooij et al. (2006) [14], which showed very promising results, with 100% of patients showing complete occlusion after embolization and surgery. Most of the studies reported recurrence rates as low, although some studies, like Sun et al. (2020) [13] and Zhou et al. (2007) [1], did not report any recurrences. On the other hand, some cohorts, like that conducted by Li et al. (2018) [11], showed a small subset of the patients who developed rebleeding that called for further intervention.

Generally, the response to treatment was good, since most of the patients either showed marked clinical improvement or had a complete resolution of symptoms. For instance, Byrne and Garcia (2013) [3] reported that three patients demonstrated complete symptom resolution while six others showed marked improvement. Similarly, Liu et al. (2014) [12] reported that almost all patients experienced significant symptomatic relief following transarterial embolization and follow-up imaging, demonstrating no rebleeding or worsening of the condition. Outcomes, as measured by the modified Rankin Scale (mRS) or Barthel Index, showed that the majority of patients returned to a good or excellent functional status; studies such as that of Lawton et al. (2008) [10] reported scores of 0-2 on the mRS in the majority of the patients.

Complications

Several of these studies mentioned complications, although generally, they were not devastating. Common complications included cranial nerve palsies, transient neurological deficits, and procedural risks such as arterial rupture or venous rupture. In the Byrne and Garcia (2013) [3] series, procedural complications were seen in four patients and included arterial dissections and microcatheter ruptures. This is evidenced in studies such as Ng et al. (2003) [6], where involvement of cranial nerves occurred in the form of trigeminal and trochlear nerve palsies in up to 21% of their patients. Importantly, no permanent neurological deficits were reported in the majority of studies, with transient complications resolving over time.

Generally, mortality rates were mostly low, with the majority of studies having no deaths directly related to the procedure. The mortality rate was 15.4% in the series by Byrne and Garcia (2013), caused by arterial dissection and infection, whereas Li et al. (2018) mentioned two deaths from cardiopulmonary arrest following rebleeding. Similarly, Wajnberg et al. (2012) [15] recorded one death due to pulmonary fibrosis unrelated to the procedure. These findings also suggest that, while complications may well occur, the overall mortality risk remains low concerning the treatment of both petrous and tentorial DAVFs.

Recurrence and Case Fatality Rates

Recurrence of DAVFs following treatment was rarely reported in this series of studies reviewed. In fact, most studies, such as Jiang et al. (2008) [9] and van Rooij et al. (2006) [14], confirmed that there were no recurrences in their patient cohorts following successful treatment. The absence of recurrence in these studies reflects the effectiveness of combining embolization and microsurgery in achieving durable occlusion. If recurrence did occur in those few cases, including those within the Stapleton et al. (2018) [4] cohort, successful long-term occlusion could result in surgical re-intervention.

The case fatality rate was low in the majority of the studies. Of these, the studies by Lawton et al. (2008) [10], Pichierri et al. (2012) [2], and Sun et al. (2020) [13] recorded no deaths of patients resulting from the treatment. The study by Byrne and Garcia (2013) [3] had the highest case fatality rate of 15.4% with two deaths; one was due to arterial dissection, while the other was from infection. On the whole, the mortality rate in the treatment of both petrous and tentorial DAVFs is not readily found, provided timely and proper intervention is applied.

Discussion

Intracranial DAVFs represent a fascinating but complex group of pathologic shunts between dural arteries and venous sinuses or cortical veins. These vascular lesions account for 10%-15% of intracranial arteriovenous malformations and are mostly present in adulthood, though they can occur in childhood with more complicated features. Many DAVFs are idiopathic, though they can arise following trauma and surgery and even as a sequela of venous sinus thrombosis. Their pattern of venous drainage is very important regarding the risk of serious complications like intracranial hemorrhage, and early diagnosis by means of high-imaging resolution is very important for adequate management and treatment [16].

The current systematic review gives a comprehensive review of the clinical characteristics, imaging modalities, treatment strategies, and outcomes in patients with petrous and tentorial DAVFs. This review synthesized data from 14 studies, given various demographic factors, clinical presentations, and their therapeutic successes and failures due to different treatment modalities. Ultimately, the results indicate that both endovascular and microsurgical treatments can be done well, but treatment has to be tailored to the particular case based on the fistula's anatomical and hemodynamic features.

Demographic Considerations: Age and Gender

The demographic data from the articles showed that petrous and tentorial DAVFs mostly affect middle-aged to older patients, with an average age of 38 to 59.8 years. While no direct causal relationship between age and the development of DAVFs has been established, this age most probably reflects a summation effect of age-related vascular changes and various risk factors, including hypertension, that may induce venous hypertension and then fistula formation. Second, it should also be mentioned that DAVFs were constantly more common in males than in females in all series; the male patients accounted for about 78% of the study populations. This male predominance is in keeping with the previous literature on DAVFs and indeed intracranial vascular malformations in general, although the underlying reasons remain speculative. This could be explained by hormonal factors, differences in lifestyle, and gender-specific variations in vascular biology; however, this is speculative and requires further research to explore this hypothesis [5,9].

Clinical Presentation: Bleeding and Neurological Deficits

In the systematic review published in 2015, Cannizzaro et al. presented evolving clinical trends in the presentation and outcomes of patients diagnosed with tentorial dural arteriovenous fistulas [17]. In the past couple of decades, fewer patients have presented with ruptured lesions, as the rates decreased from 64.4% to 43.6%. This decline likely derives both from improved diagnostic capabilities, including increased use of noninvasive angiography and better recognition of venous hypertension as a key factor in neurological symptoms, but also from the following: these trends have translated into higher rates of early detection and improved overall neurologic outcomes for patients.

Clinical presentation variability is one of the most important factors to be considered in the management of DAVFs. Most patients in reviewed studies presented with symptoms ranging from headaches and tinnitus to life-threatening intracranial hemorrhages. Intracranial hemorrhage was one of the more common serious manifestations, especially in series that report tentorial DAVFs, in which as many as 50% of patients presented with subarachnoid hemorrhage [1,6]. They elevate the risk of rupture and hemorrhage with such fistulas present, pointing out the aggressive nature in their natural history when cortical venous drainage manifests.

Other nonbleeding neurological deficits included cranial nerve palsies, vertigo, and ataxia and were not infrequent, particularly in fistulas that were located near structures of cranial nerves. This is indicative of the compressive and ischemic effects brought about by venous congestion as a result of abnormal arteriovenous shunting. Patients who presented with symptoms other than bleeding usually had a better prognosis after treatment, specifically if diagnosed and treated before structural neurological damage had occurred [2,10].

Imaging Modalities: DSA as the Gold Standard

Accordingly, this review confirms DSA as the diagnostic gold standard in the investigation of DAVFs because it provides highly detailed visualization not only of arterial feeders and venous drainage patterns but also of the precise location of the fistula. In addition, all studies reported the universal application of DSA not only at diagnosis but also in post-treatment follow-up, indicating its great help in guiding therapeutic decisions [3,14].

Although DSA remains the most sensitive imaging modality regarding DAVFs, these non-invasive techniques of MRI and CT were useful in the detection of hemorrhage and the determination of venous drainage patterns before angiography. MRI was especially useful for the identification of venous anomalies like varices and thrombosed veins and the detection of associated parenchymal changes due to venous congestion, such as edema or gliosis [4,11]. In patients with suspected hemorrhage, CT helped in diagnosing acute bleeds, mainly subarachnoid and intraventricular hemorrhages, which were common in tentorial DAVFs [1].

Emerging imaging modalities, including indocyanine green video angiography, have been very encouraging in an intraoperative role. Sun et al. (2020) [13] illustrated the use of ICG-VA in the course of microsurgery to confirm complete obliteration of the fistula. The development of such a real-time intraoperative imaging modality has afforded the surgeon clear intraoperative visualization of blood flow dynamics in vessels and minimal need for postoperative angiography. With advances in technology, the integration of real-time intraoperative imaging might one day become routinely incorporated into the treatment of complex DAVFs.

Treatment Modalities: Endovascular vs. Microsurgical Approaches

The management of petrous and tentorial DAVFs is particularly challenging because these lesions are situated in different anatomical locations with diverse hemodynamic characteristics. This systematic review has shown that endovascular and microsurgical approaches are both effective in the treatment of these vascular lesions, although success and applicability depend greatly on the individual patient and fistula characteristics. In some instances, a combined approach that leverages the strengths of both modalities offers the best opportunity for durable fistula obliteration and symptom resolution.

Endovascular Treatment Trials

In the management of cranial dAVFs, endovascular approaches have become the first line of treatment because they are the least invasive and enjoy a very high success rate. Advanced materials like Onyx and Squid show great promise for complete occlusion of the fistula with preservation of normal venous drainage in techniques such as transarterial and transvenous embolization. The surgical approach is conserved for complicated cases, mainly because of the failure of the endovascular approach; however, comprehensive and individualized treatment can be offered by surgery [18].

When accessible arterial feeders are present, endovascular treatment has become the mainstay of management in DAVFs. Huang et al. (2009) [8] and Liu et al. (2014) [12] described transarterial embolization using Onyx as a highly effective obliteration rate reaching up to 78% and 92%, respectively, within their aggregate series. The liquid embolic agent Onyx allows a finer approach to an embolization procedure, and as it is cohesive, it minimizes the possibility of nontarget embolization. Wajnberg et al. (2012) [15], meanwhile, took this a step further by using both transarterial and transvenous embolization to show flexibility for endovascular approaches combined with access into the venous system. The benefits of minimal invasiveness reduce the patient's recovery time and limit the procedural complications associated with open surgery. Jiang et al. (2008) [9] also proved the benefits of endovascular treatment using transarterial and transvenous embolization. In their cohort of 19 patients, 13 were able to be completely obliterated from the fistula, while the remaining six had residual fistulas developing for which additional treatment was necessary. The combined use of both transarterial and transvenous approaches proved to be particularly important for the complete cure in the most difficult cases, where embolization via arterial access alone failed. However, complications such as rupture of the veins and palsies in cranial nerves are well-known risks of endovascular techniques, especially when accessing venous structures. This study therefore pointed out the need for considering both arterial and venous approaches in cases with complex fistula anatomy.

Nevertheless, even though endovascular techniques are very effective in cases with an accessible arterial supply, they are deficient in certain scenarios, especially when the arterial anatomy is more complex. These demonstrate that while the endovascular technique is minimally invasive, it does not always provide a complete or long-term solution in more complex lesions. The need for repeat embolizations or additional interventions is also a potential drawback in some studies.

Microsurgical Treatment Studies

Although more invasive, microsurgical techniques also offer advantages, particularly for DAVFs with complex venous drainage and arterial supply not easily accessible via endovascular routes. Lawton et al. (2008) [10] and Hatano et al. (2013) [5] reported high success rates with the use of microsurgical interruption of venous drainage, achieving complete obliteration in 94% and 100% of patients, respectively. The advantage of microsurgery lies in the ability to directly visualize and disconnect the fistula, providing a more definitive treatment, especially in cases where endovascular approaches fail. Microsurgery is effective, particularly for deep-seated lesions or those with complicated arterial feeders where embolization may fail either to reach or adequately occlude the fistula. Hatano et al. (2013) [5] employed subtemporal and supracerebellar approaches, allowing precise control over the vascular anatomy, particularly in the tentorial region, where critical neurovascular structures are present.

However, all microsurgical approaches carry risks. Most studies, such as those by Lawton et al. (2008) [10] and Sun et al. (2020) [13], have reported low long-term morbidity, but craniotomy and manipulation of sensitive structures still carry significant risks. Sun et al. (2020) [13] demonstrated the precision of intraoperative guidance by ICG-VA in microsurgical techniques, with no recurrence in their patient cohort. This underscores the expertise required for successful microsurgical intervention. When performed by skilled teams, microsurgery offers a more durable solution with a lower likelihood of recurrence, particularly for complex DAVFs that are not amenable to embolization.

Combined Endovascular and Microsurgical Treatment Studies

Multiple reports have highlighted the value of combining endovascular and microsurgical approaches, maximizing the strengths of each modality while minimizing their limitations. Stapleton et al. (2018) [4] demonstrated the utility of this approach, where patients initially treated with embolization for superior petrosal sinus DAVFs required surgery for recurrence. The combined approach resulted in excellent long-term outcomes, with a 100% surgical cure rate. Similarly, Ng et al. (2003) [6] and Li et al. (2018) [11] reported success in complex cases where embolization alone was insufficient. In such cases, endovascular treatment reduces the complexity of the fistula by decreasing its size and arterial supply before surgery, making the subsequent microsurgical procedure less invasive and more likely to succeed. Byrne and Garcia (2013) [3] demonstrated the value of a combined approach, using both endovascular embolization and microsurgical intervention. Five patients were cured with embolization alone, while others required surgery to achieve complete obliteration. This staged approach reduced fistula complexity before surgery, highlighting the benefit of combining techniques in more challenging cases.

The combination approach offers a compelling solution for refractory cases and complex fistulas with multiple arterial feeders or complicated venous drainage patterns. By embolizing some arterial feeders, the surgical complexity is reduced, lowering the risks associated with direct surgical intervention. This approach also allows for tailored treatments, where decisions can be made based on intraoperative findings, using both embolization and microsurgery as needed. For instance, Li et al. (2018) [11] demonstrated that the initial embolization, even if incomplete, reduced the size and blood flow through the DAVF, enabling microsurgical excision to be more controlled and less risky.

The review of these studies reveals a nuanced understanding of how DAVFs should be approached. Endovascular treatment is highly effective in cases with straightforward arterial anatomy, especially when embolic agents like Onyx are used. It offers a minimally invasive option with good outcomes in many cases, as seen in Huang et al. (2009) [8] and Liu et al. (2014) [12]. However, as illustrated by Li et al. (2018) [11] and Wajnberg et al. (2012) [15], its limitations become apparent in cases where arterial access is complex or incomplete obliteration is achieved, necessitating further intervention.

On the other hand, microsurgery provides a more definitive solution, particularly in cases where embolization is not feasible or fails. Lawton et al. (2008) and Hatano et al. (2013) highlight how direct interruption of venous drainage or coagulation of the fistula offers a high rate of success with durable outcomes. Yet, the higher risk profile and invasiveness of surgery make it less appealing as a first-line treatment unless the fistula is particularly complex [5,10].

Ultimately, the most compelling approach, as demonstrated by studies such as Stapleton et al. (2018) [4] and Ng et al. (2003) [6], appears to be a combined approach. This allows physicians to utilize the advantages of both endovascular and microsurgical techniques, treating the most accessible aspects of the fistula with embolization and addressing the more difficult-to-reach areas with surgery. By combining these approaches, patients benefit from the minimally invasive nature of endovascular techniques while still receiving the definitive care that microsurgery offers in more complex cases.

Going forward, the optimal management of petrous and tentorial DAVFs should be highly individualized. Endovascular approaches should be the first-line treatment in accessible and less complex cases. For more complex DAVFs, especially those involving multiple feeders or deep venous drainage, combining embolization with surgery offers the most durable and effective treatment option. Further research is needed to refine treatment algorithms, particularly for determining when to employ a combined approach from the outset.

Complications and Case Fatality Rates

A number of studies reported complications; these were generally manageable, however, with treatments being tailored to fistula complexity and patient condition. Common complications included cranial nerve palsies, transient neurological deficits, and procedural risks such as arterial or venous rupture. In the paper by Byrne and Garcia (2013) [3], serious procedural complications during endovascular embolization included four patients who developed arterial dissection and microcatheter rupture. Two of these cases resulted in deaths, one due to arterial dissection and the other due to infection. These findings definitely underpin the fact that while the endovascular techniques are minimally invasive, they are not devoid of serious risks, especially in processes yielding complicated fistulas with venous access or repeated embolization approaches. Jiang et al. (2008) [9] also reported rupture of veins and cranial neuropathies and showed that approaching the venous drainage through endovascular accessibility can lead to specific complications.

While more invasive, microsurgical techniques tended to report fewer serious complications in the hands of experienced teams. Lawton et al. (2008) [10] and Hatano et al. (2013) [5] reported transient neurological deficits using microsurgical approaches without permanent damage or treatment-related death. Sun et al. (2020) [13], with step braking and advanced imaging guidance like ICG-VA, also showed excellent outcomes without recurrence or mortality. This would suggest that, in selected complex DAVFs, microsurgery not only carries a higher definitive cure but may also be associated with a lower major complication rate. Of course, the key factor is that surgery is performed by a team that is experienced in such technical procedures.

The combined approach thus may be expected to carry slightly higher complication rates, considering the nature of cases treated: more complex. Stitching complications such as cranial nerve IV palsy were noted in one case by Stapleton et al. (2018) [4] undergoing surgery for their recurrent DAVFs, usually primarily treated with embolization. The recurrence itself underlines the limitation of endovascular approaches in more complicated anatomical cases. This enhances the importance of combining embolization with microsurgery for a more durable result. It is reassuring, though, to note that such complications were transient and resolved with time, with no permanent neurological deficits recorded. Ng et al. (2003) [6] also reported temporary cranial nerve palsies after the combined treatment; these patients, however, also recovered full function eventually. These studies thus provide indications that additional risks may be introduced with combined approaches, especially for more complex conditions, but generally are often associated with good long-term results, especially where initial endovascular treatment is insufficient.

The case fatality rate was generally very low for most of the studies and reflected the relative safety of these interventions when performed by experienced teams. The highest case fatality rate was Byrne and Garcia (2013), with two deaths out of 13 patients (15.4%) attributed to arterial dissection and infection during the endovascular phase of treatment. This case fatality rate underlines the serious risks of multiple or complex embolization procedures when venous access is needed or in cases with arterial dissections. Indeed, most studies, such as those by Lawton et al. (2008) [10], Wajnberg et al. (2012) [15], and Sun et al. (2020) [13], reported no deaths related to the treatment, demonstrating indeed that by cautious selection of the modality of treatment, mortality can almost be completely avoided.

Data from these studies suggest that both approaches carry their specific risks, but complications can be kept to a minimum, provided there is judicious patient selection and an individualized treatment plan. For example, the higher complication rates associated with venous access in endovascular procedures, as noted in the study by Jiang et al. (2008) [8] and Byrne and Garcia (2013) [3], emphasize the importance of very careful planning, particularly in those patients with fistulas exhibiting complicated patterns of venous drainage. Further refinement of patient selection for endovascular approaches in the future may be guided by perhaps limiting these to fistulas with simpler arterial access and referring more complex cases to combined or microsurgical approaches.

Moreover, the successes of microsurgical techniques in reducing long-term complications in both Lawton et al. (2008) [10] and Sun et al. (2020) [13] give validity to a statement that microsurgery may be preferable in high-risk cases, particularly with complex fistula anatomy, or after failed embolization. The intraoperative imaging modality, such as ICG-VA, provides an added layer of precision to reduce the possibility of recurrence and the need for further intervention.

Lastly, combined approaches, while having a higher early complication rate due to the complexity of cases treated, seem to have long-term benefits outweighing these risks. A combined approach allows presumably safe surgical resection in cases where embolization has shrunk the fistula and its complexities, as was demonstrated by Stapleton et al. (2018) [4] and Ng et al. (2003) [6]. This would suggest that, when premeditated, this combination can significantly improve the outcomes in very complex or recurrent DAVFs. Future directions may involve more standardized protocols to identify early in treatment for finding the right time for a combined approach rather than as a salvage option after embolization failure.

In the 2024 study, Meshari emphasizes the complexity of managing medial tentorial DAVFs, highlighting the critical role of multidisciplinary approaches. These rare but dangerous lesions demand prompt intervention due to their high risk of hemorrhage and neurological complications. The case demonstrated how initial endovascular embolization, although effective, can be complicated by residual fistulas and venous hypertension, necessitating vigilant follow-up. This underscores the importance of combining endovascular and surgical techniques to ensure comprehensive treatment and prevent life-threatening outcomes [19].

New Insights and Future Directions

In their 2024 case report, Sugihara et al. [20] documented a rare but impactful example of managing a tentorial dural arteriovenous fistula that initially resisted complete embolization with Onyx. By employing a "bailout" strategy with n-butyl-2-cyanoacrylate (NBCA), they achieved complete obliteration of the fistula. This case highlights the importance of combining different embolic agents to address incomplete outcomes, illustrating a significant advancement in endovascular techniques for high-risk vascular lesions. The strategic use of Onyx and NBCA exemplifies how modern neurosurgical interventions are evolving to improve success rates in even the most challenging cases. In a 2021 case report, Panagiotopoulos et al. [21] detailed a groundbreaking intervention for a ruptured venous ectasia linked to a Cognard IV tentorial DAVF. When conventional transarterial access failed due to vessel tortuosity, the team used ultrasound-guided transjugular embolization, successfully navigating the complex venous architecture and deploying coils to seal the rupture. This approach not only controlled the immediate hemorrhage but also preserved the venous outflow, allowing for later stereotactic radiosurgery. The case highlights how ultrasound guidance can transform the management of high-risk vascular emergencies, enabling precise and life-saving interventions.

The findings from this systematic review highlight several important considerations for the future management of DAVFs. First, the importance of early diagnosis cannot be overstated. As seen in studies with longer symptom duration, delays in treatment were associated with poorer outcomes and greater neurological morbidity. Second, while endovascular techniques have advanced significantly, they are not always curative, particularly in fistulas with complex arterial supply. In such cases, a hybrid approach that combines embolization and microsurgery may offer the best chance of durable fistula obliteration.

Looking ahead, further research is needed to refine patient selection criteria for each treatment modality. The role of advanced imaging techniques, such as ICG-VA, should also be explored in larger cohorts, as real-time intraoperative imaging could enhance surgical precision and reduce recurrence rates. Finally, as newer embolic agents and devices are developed, future studies should evaluate their efficacy in comparison to Onyx, particularly in fistulas fed by the internal carotid artery.

## Conclusions

The complex anatomy and disastrous neurological sequelae make the management of petrous and tentorial DAVFs clinically challenging. Therefore, this review highlights that endovascular and microsurgical treatments are undeniably effective; however, treatment for each fistula should be individualized according to the given characteristics. Transarterial Onyx embolization represents an excellent, less invasive alternative, especially in those with accessible arterial feeders. However, given the risks of complications, it may not be sufficient for more challenging cases. In contrast, microsurgical approaches constitute a more durable form of cure, particularly fistulas associated with an intricate arterial supply or deep venous drainage, with low complication rates when performed by experienced teams.

Complex and recurrent DAVFs are best treated by combined strategies. This two-pronged approach maximizes outcomes and minimizes recurrence. These results will be further enhanced in the future with improved patient selection and judicious use of advanced imaging techniques. Early aggressive multimodal therapies in complex cases may reduce the need for re-intervention, thus achieving higher long-term success rates in the management of petrous and tentorial DAVFs.
